# Supplementary figures and images for: Astrocytes promote a protective immune response to brain Toxoplasma gondii infection via IL-33-ST2 signaling
Source: PLoS Pathog. 2020 Oct 27;16(10):e1009027. doi: 10.1371/journal.ppat.1009027 (PMC7647122; doi:10.1371/journal.ppat.1009027)

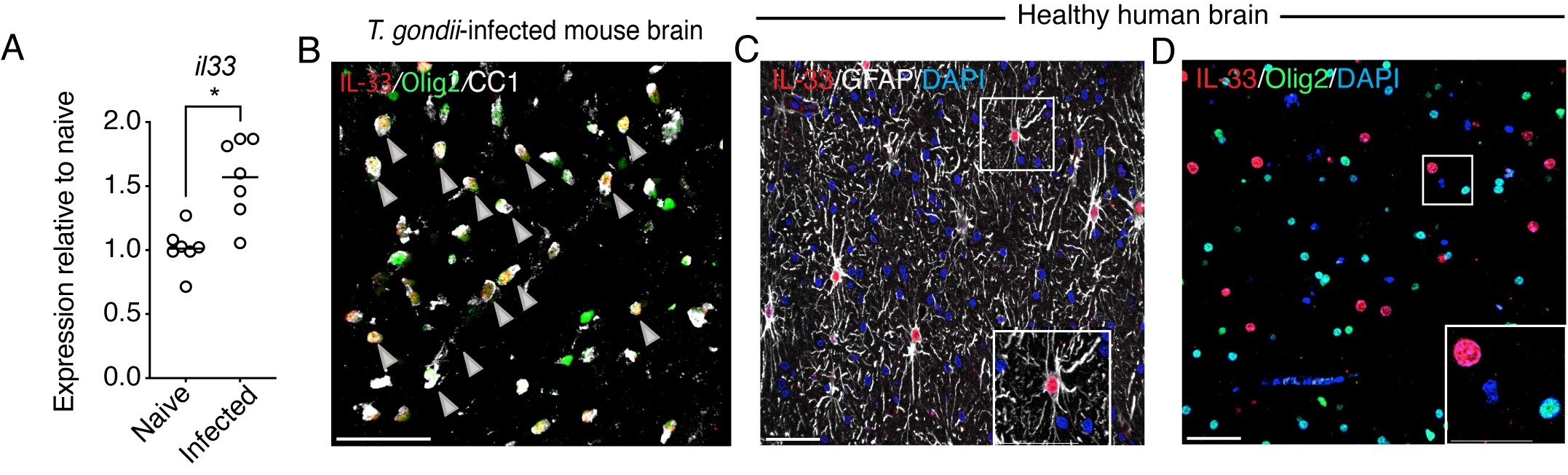

Supplement: S1 Fig — (A) Real time PCR for il33 transcript from whole brain homogente at 4 weeks post infection compared to naïve brain tissue. (B) Colocalization, denoted by gray arrows, of nuclear IL-33 protein (red) with mature oligodendrocytes, marked by nuclear Olig2 expression (green), and CC1(white) by confocal fluorescence microscopy of infected mouse brain tissue. (C and D) Confocal fluorescence microscopy of nuclear IL-33 stain present in astrocytes (C) but not oligodendrocytes (D) in the temporal lobe of human brain tissue from patients that did not succumb to toxoplasmic encephalitis (healthy). Statistical significance was determined by randomized block ANOVA (A), which shows data pooled from two independent experiments * = p < .05, ** = p < .01, *** = p < .001. Scale bars indicate 50μm. (TIF) [file ppat.1009027.s001.tif]

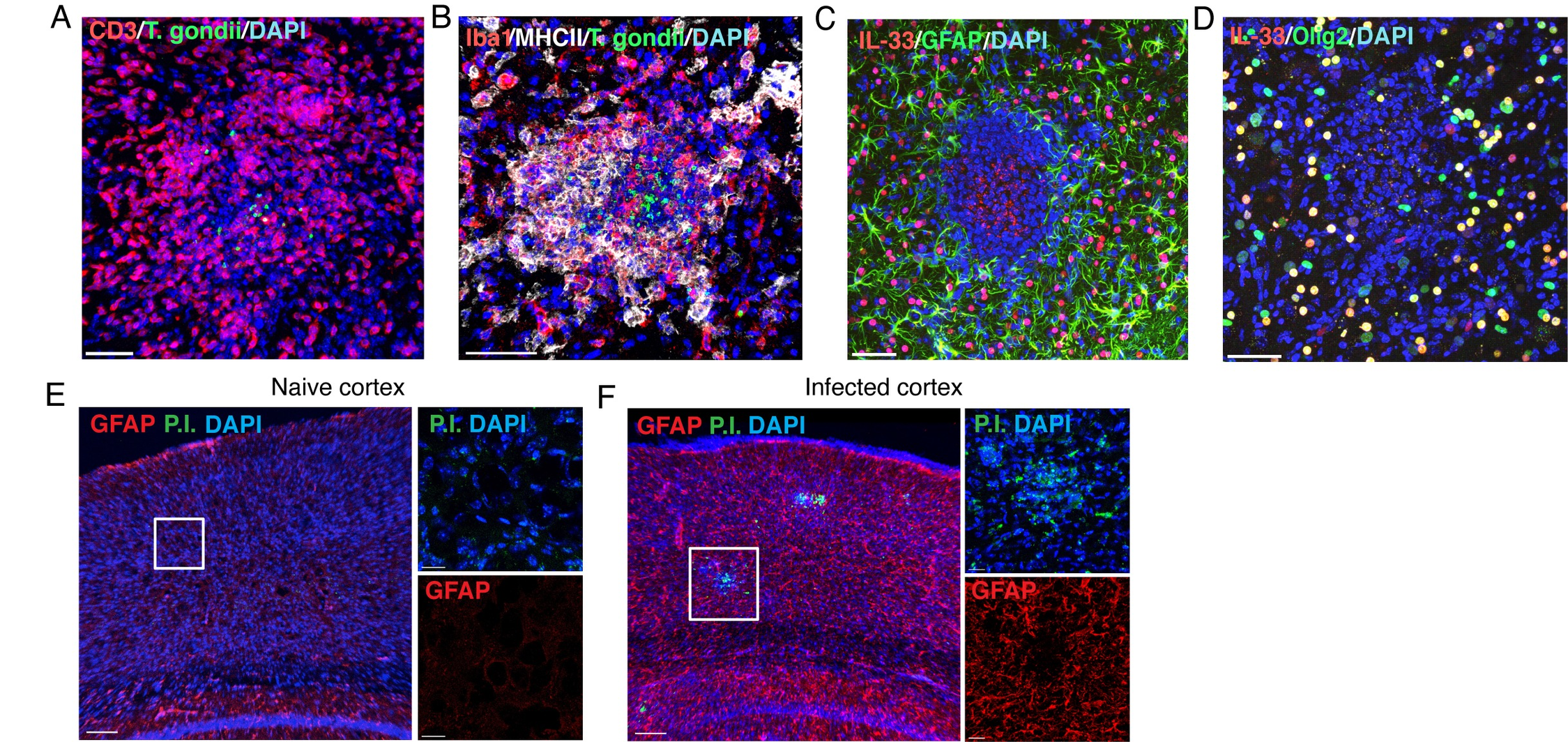

Supplement: S2 Fig — (A and B) Representative images of immune cells surrounding foci of individual replicating parasites (green) in cortical brain tissue, including CD3+ T cells (red) (A), and MHCII+ (white) Iba1+ (red) myeloid cells (B). (C and D) Representative images of necrotic foci, featuring a loss of brain resident cells which express IL-33 (red), including GFAP+ astrocytes (green) (C), and Olig2+ oligodendrocytes (green) (D). (E and F) Representative images of propidium iodide fluorescence in brain tissue, 24 hours post i.p. injection into naïve and infected mice. Staining depicts propidium iodide (green), and GFAP+ astrocytes (red). Scale bars indicate 50μm in A-D, and 100μm or 30μm (insets) in E and F. (TIF) [file ppat.1009027.s002.tif]

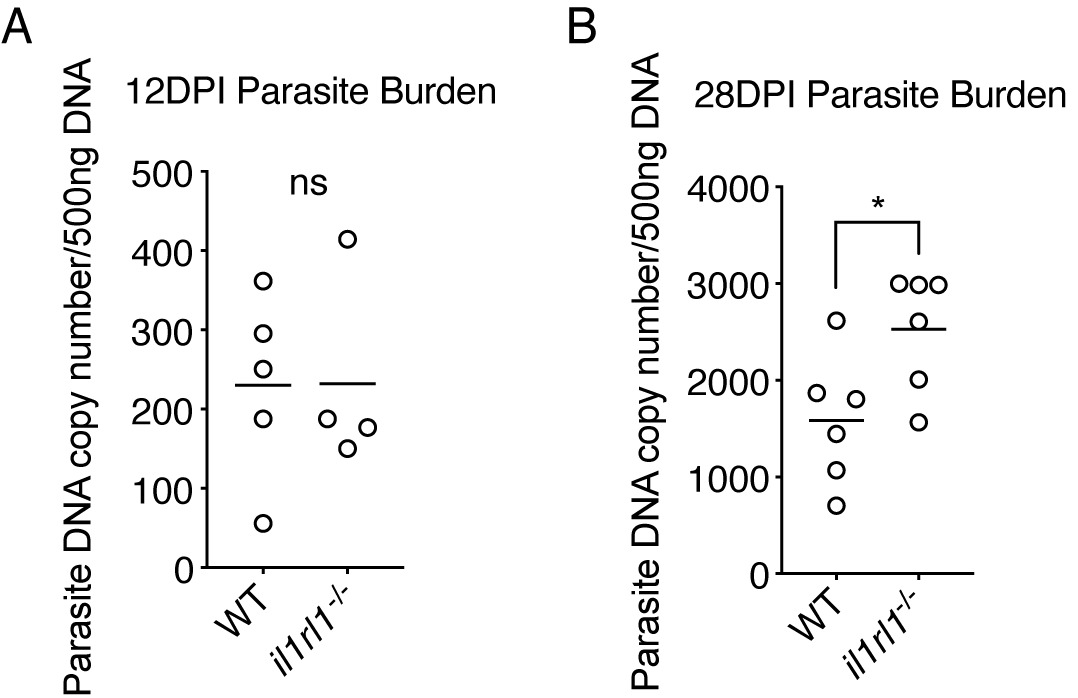

Supplement: S3 Fig — (A and B) Real time PCR for parasite genomic DNA from whole-brain homogenate of infected WT and il1rl1-/- mice at 12 days post infection (DPI) (A) and 28DPI (B). Statistical significance was determined by two tailed t-test (A) or a randomized block ANOVA (B), which shows data pooled from two independent experiments * = p < .05, ** = p < .01, *** = p < .001. (TIF) [file ppat.1009027.s003.tif]

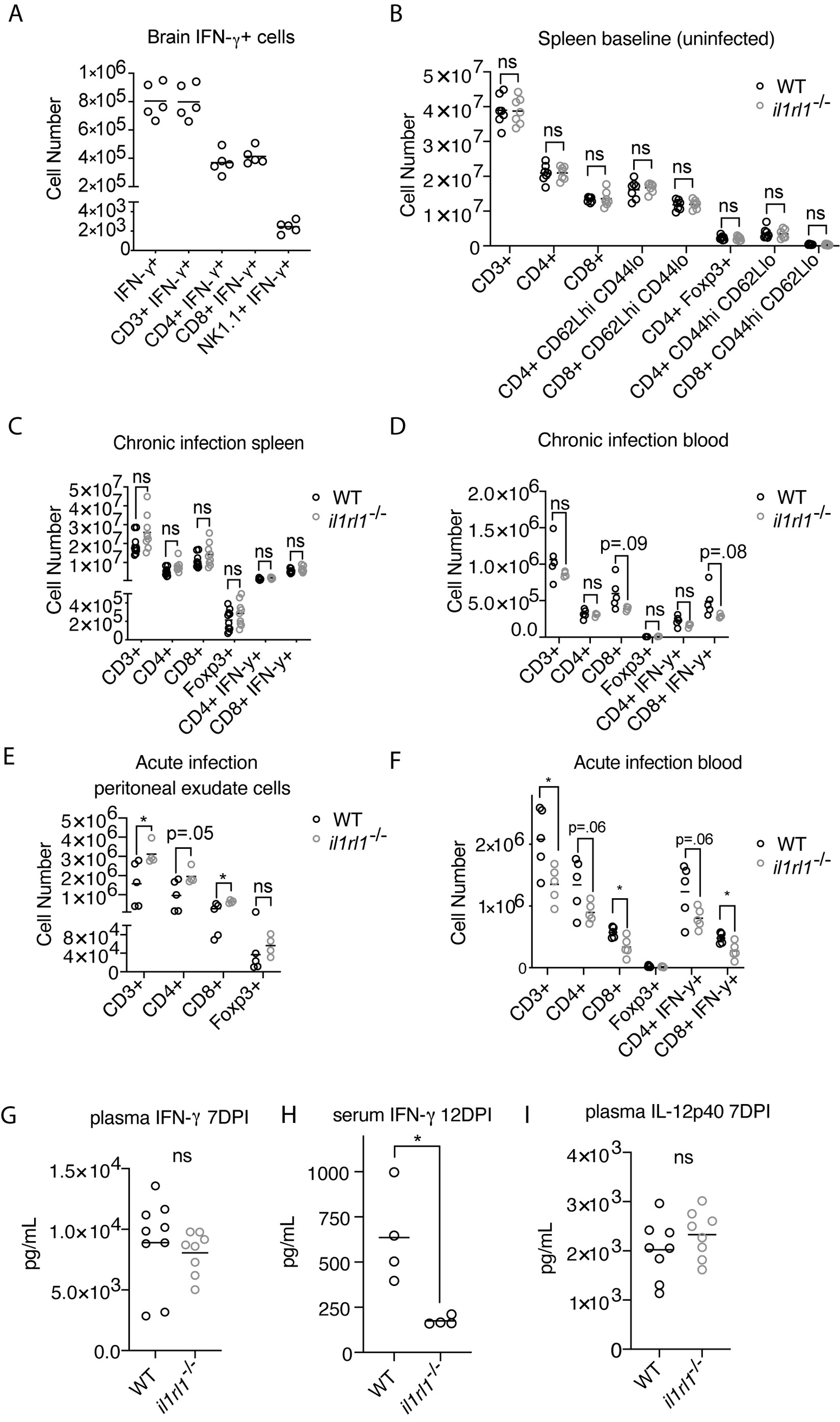

Supplement: S4 Fig — (A) Breakdown of total IFN-γ+ cells by cell type by flow cytometry in infected brain tissue four weeks post infection. IFN-γ was measured following stimulation ex vivo for five hours with PMA/ionomycin. (B) Assessment of spleen T cell numbers in il1rl1-deficient mice prior to infection by flow cytometry. (C and D) assessment of peripheral tissue T cell numbers and activation, including spleen (C) and blood (D) by flow cytometry 4 weeks post infection. (E and F) T cell numbers at day 10 acute infection by flow cytometry in the peritoneum (E) and blood (F). (G-I) plasma (G,I) or serum (H) ELISAs for IFN-γ (G,H) or IL-12 (I) during acute infection. Statistical significance was determined by randomized block ANOVA when two experiments were pooled (B,C, G, I), or by two-tailed t-test (D, E, F, H) * = p < .05, ** = p < .01, *** = p < .001. (TIF) [file ppat.1009027.s004.tif]

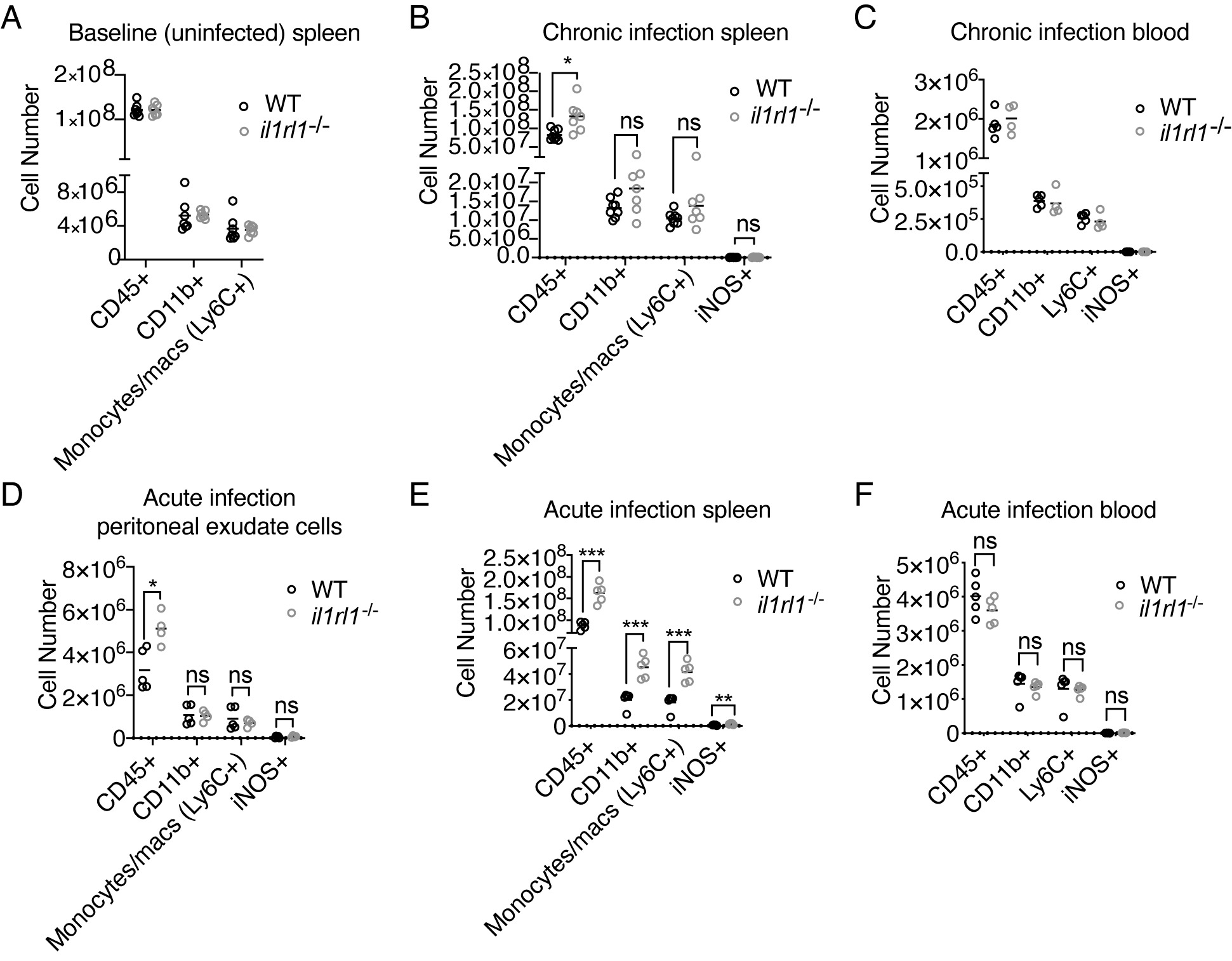

Supplement: S5 Fig — (A) Assessment of spleen myeloid cell numbers in il1rl1-deficient mice prior to infection by flow cytometry. (B and C) assessment of peripheral tissue myeloid cell numbers and activation, including spleen (B) and blood (C) by flow cytometry 4 weeks post infection. (D-F) Myeloid numbers at day 10 during acute infection by flow cytometry in the peritoneum (D), spleen (E) and blood (F). Statistical significance was determined by randomized block ANOVA when two experiments were pooled (A and B), or by two-tailed t-test (C-F) * = p < .05, ** = p < .01, *** = p < .001. (TIF) [file ppat.1009027.s005.tif]

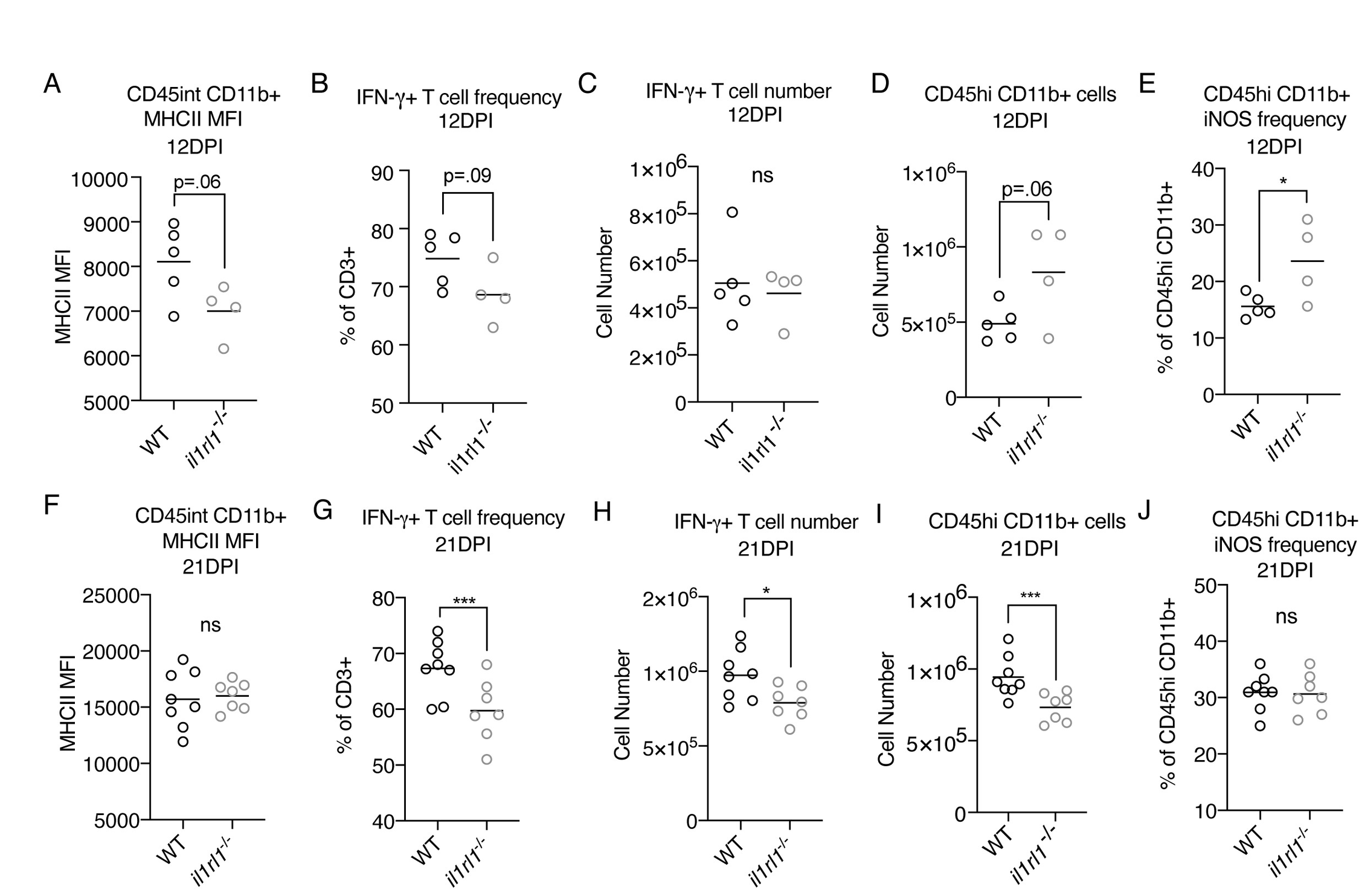

Supplement: S6 Fig — (A-J) Assessment of T cell and myeloid cell number and activation in the brain by flow cytometry at 12 days post infection (12DPI) (A-E) or 21 days post infection (21DPI) (F-J). Statistical significance was determined by two-tailed t-test (A-E), or by randomized block ANOVA when two experiments were pooled (F-J) * = p < .05, ** = p < .01, *** = p < .001. (TIF) [file ppat.1009027.s006.tif]

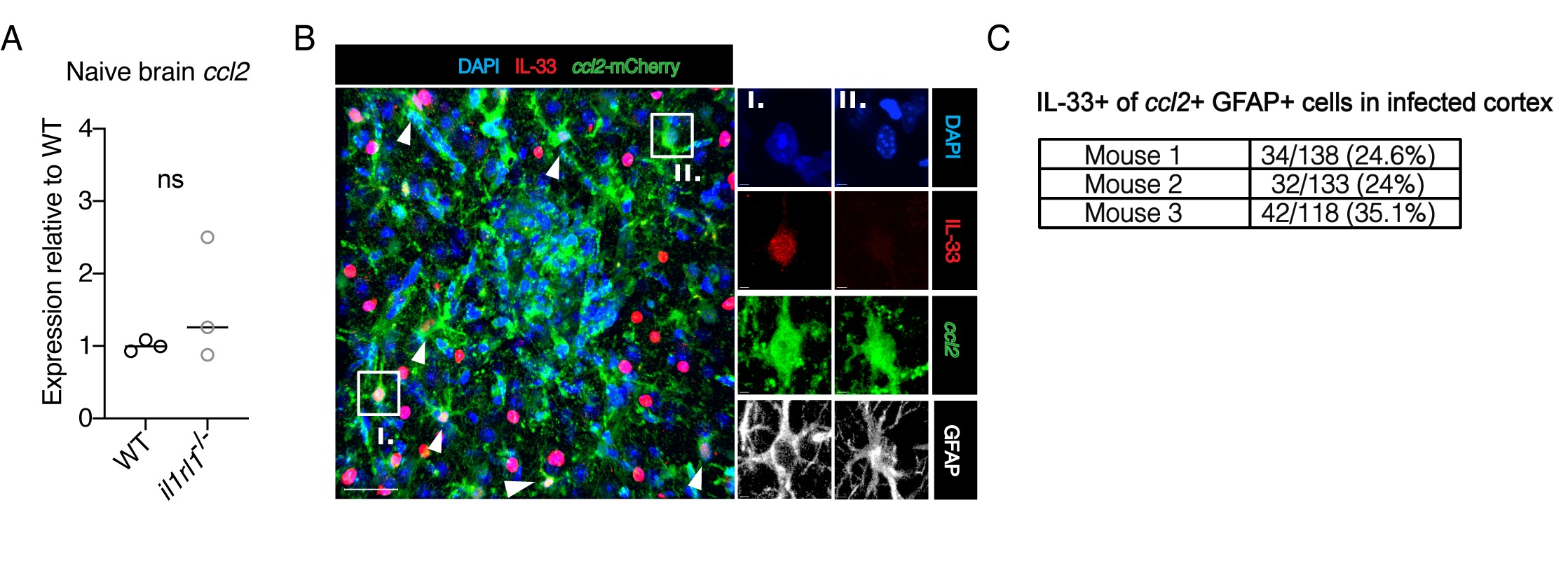

Supplement: S7 Fig — (A) Real-time PCR analysis of whole-brain ccl2 expression in naïve WT and il1rl1-/- mice. (B) Colocalization, denoted by white arrowheads, of nuclear IL-33 protein (red) with ccl2 (green), both expressed by GFAP+ astrocytes (insets, white) by confocal fluorescence microscopy. (C) Quantification of frequency of colocalization of IL-33 and ccl2 in cortical astrocytes. Statistical significance was determined by two-tailed t-test (A) * = p < .05, ** = p < .01, *** = p < .001. Scale bars indicate 30μm and 3μm(A, insets). (TIF) [file ppat.1009027.s007.tif]

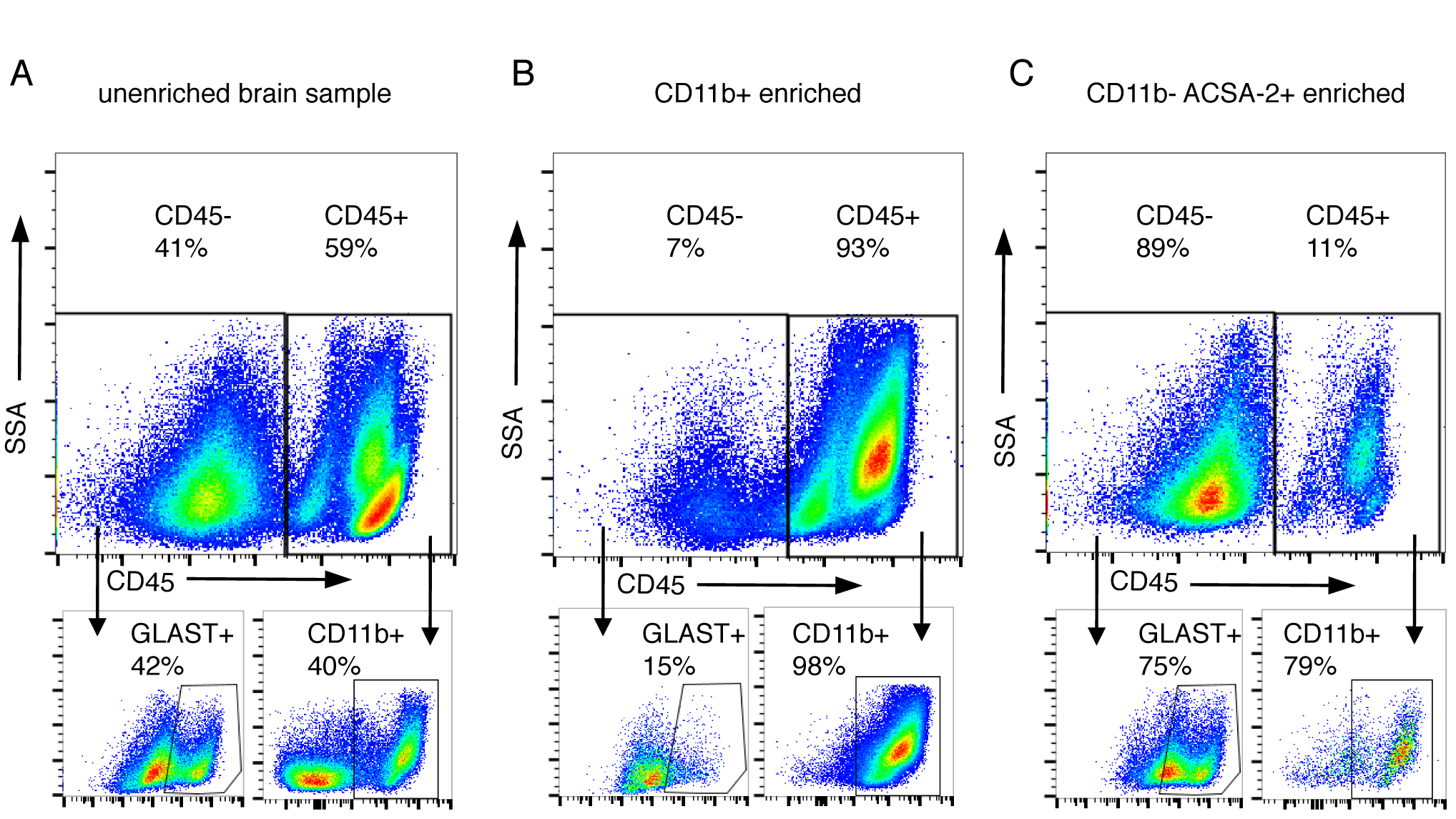

Supplement: S8 Fig — (A) Unenriched single cell suspension of all purified cells from infected brain tissue 4 weeks post infection. (B and C) Assessment of purity achieved by enriching for myeloid cells using CD11b+ magnetic beads (B), or astrocytes (C), by negatively selecting for myeloid cells using CD11b+ magnetic beads, followed by positive selection for astrocytes with ACSA-2+ magnetic beads. (TIF) [file ppat.1009027.s008.tif]

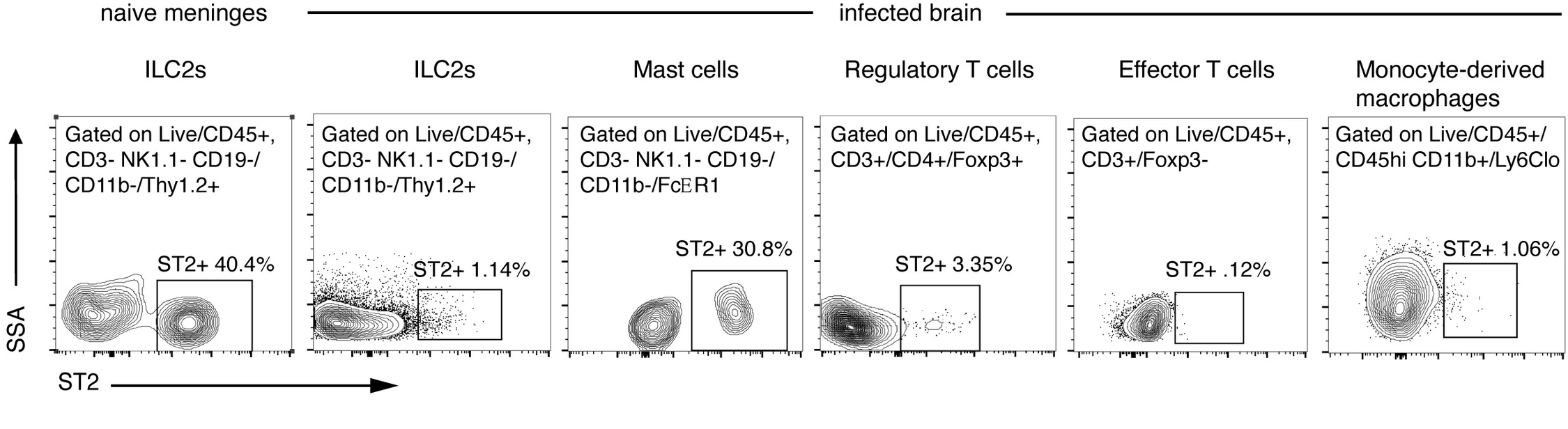

Supplement: S9 Fig — Detection of ST2 expression by flow cytometry of type 2 innate lymphoid cells, mast cells, regulatory T cells, effector T cells, and monocyte-derived macrophages in 4wk T. gondii-infected brain tissue. (TIF) [file ppat.1009027.s009.tif]

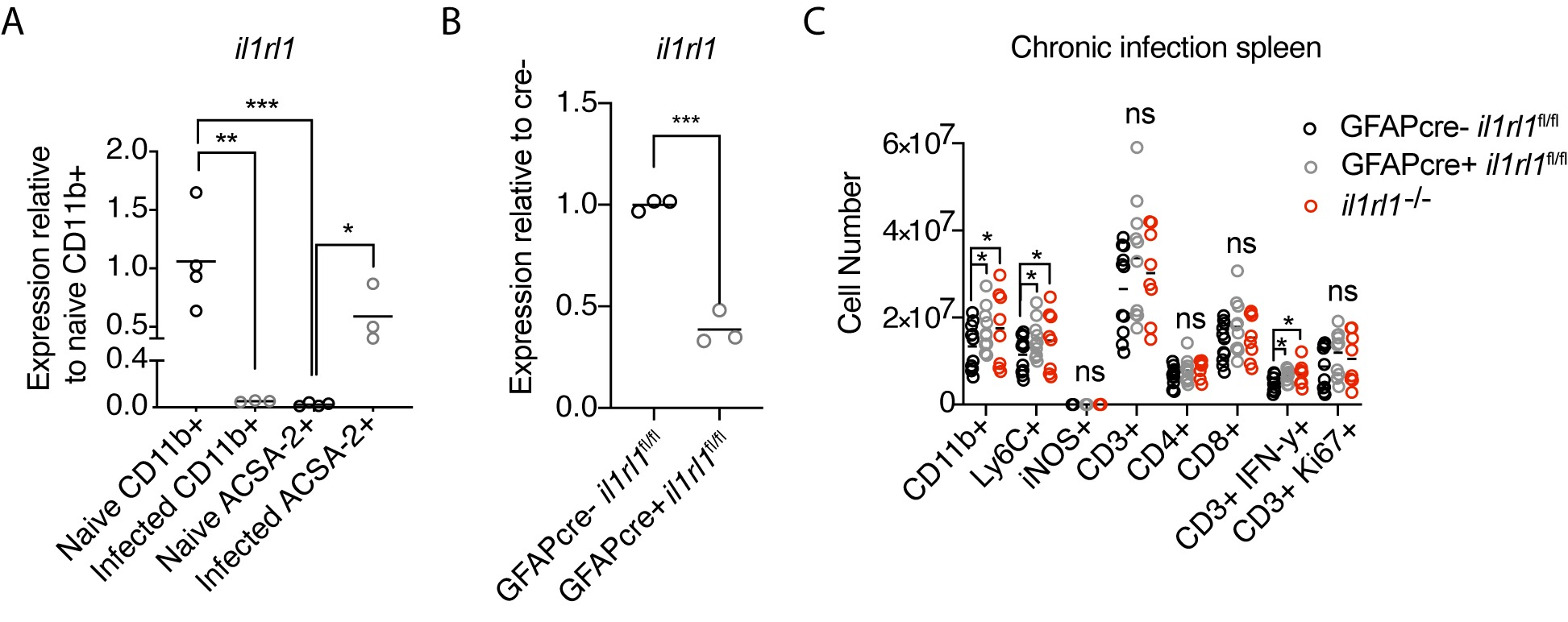

Supplement: S10 Fig — (A) Cell-type specific magnetic enrichment for myeloid cells (CD11b+) or astrocytes (CD11b- and ACSA-2+) in naïve and chronically infected brain tissue. Single cell suspensions of enriched cells were resuspended in Trizol, RNA extracted, and measured by real time PCR for ilrl1(st2) expression. (B) Validation of excision of il1rl1 from magnetically-enriched astrocytes in GFAPcre il1rl1fl/fl mice by quantitative PCR (C) Assessment of spleen immune cell numbers by flow cytometry four weeks post infection. Statistical significance was determined by one-way ANOVA with Tukey’s test (A), a two-tailed t-test (B), or a randomized block ANOVA (C) * = p < .05, ** = p < .01, *** = p < .001. (TIF) [file ppat.1009027.s010.tif]

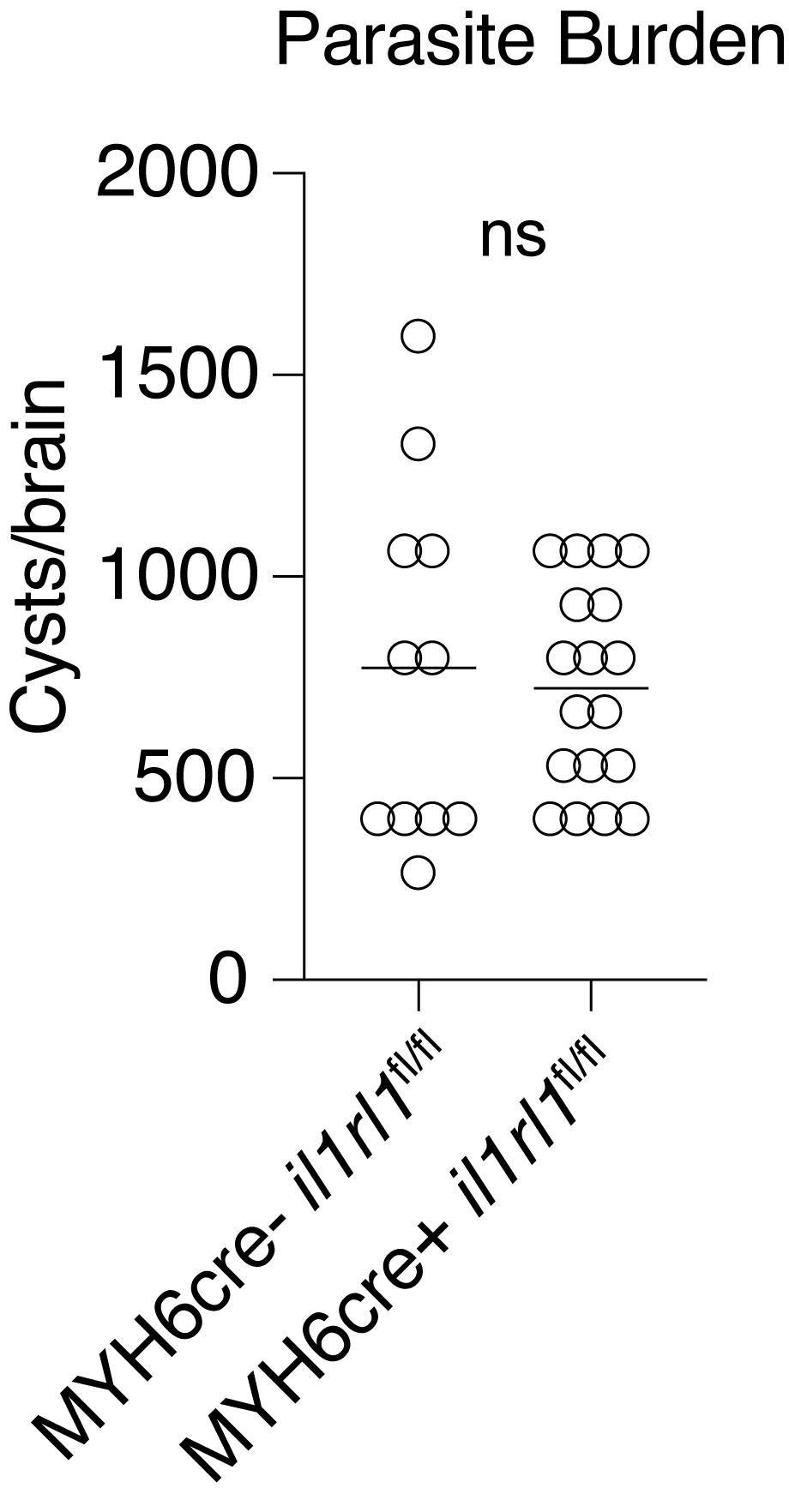

Supplement: S11 Fig — Parasite burden as measured by cyst count from brain homogenate. Statistical significance was determined by randomized block ANOVA. (TIF) [file ppat.1009027.s011.tif]
