# Supplementary material for: Astrocytes promote a protective immune response to brain Toxoplasma gondii infection via IL-33-ST2 signaling
Source: PLoS Pathog. 2020 Oct 27;16(10):e1009027. doi: 10.1371/journal.ppat.1009027 (PMC7647122; doi:10.1371/journal.ppat.1009027)
Supplement: Attachment — Submitted filename: Still_Plos Pathogens rebuttal 9-1-20.docx [file ppat.1009027.s013.docx]

Reviewer Comments (if any, and for reference):

Reviewer's Responses to Questions:


Reviewer #1:

1. Stimulation of IL-33: Although a role for IL-33 in the CNS during disease has been established including during chronic Toxoplasma infection, the source and targets for this cytokine have not. The authors rightly point to the initial sensing of infection as a gap in our knowledge. IL-33 is presumably released following DAMP recognition however although referred to in the discussion evidence of necrosis has not been provided. Beautiful immunohistochemistry clearly shows the nuclear localization of IL-33 and the lack of it around areas of inflammation. Negative areas of IL-33 could be nuclear release or it could be changes in cellular composition in inflammatory foci. Staining for necrosis in these areas would help to establish the presence of IL-33-releasing DAMPs or suggest that necrosis is not the likely cause as also postulated in the discussion.

We thank the reviewer for this comment since we are also very interested in how IL-33 is released in the infected brain. To understand if cell death could be occurring in infected brain tissue and therefore could be responsible in part for IL-33 release, we injected propidium iodide intraperitoneally into naïve and chronically infected mice and imaged their brains 24 hours later (**Figure S2E and F**). We used propidium iodide since it is not limited to detecting apoptosis, such as cleaved caspase 3 or TUNEL staining, but should instead enter any dying cell with a loss of membrane integrity. We found that inflammatory foci in infected brains, denoted by DAPI clustering and absence of GFAP staining, often contained a high degree of propidium iodide positivity, compared with a lack of positivity in naïve brains (**Figure S2E and F**). These results suggest that some form of cell death is occurring in areas of inflammation in infected brain tissue, which could play a role in releasing IL-33, although the possibility that IL-33 is secreted from living cells cannot be eliminated. These results are discussed in **lines 158-164 on page 7**.

2. Source of IL-33: In Figure 1 strong evidence demonstrates oligodendrocytes as an important source of IL-33 while the measurement of IL-33 in the CSF is particularly impressive. GFAP and IL-33 overlap is not as convincing most likely due to the less than satisfying structural GFAP versus nuclear IL-33 discreet locations. Do in vitro cultured astrocytes produce IL-33? The authors provide an important piece of information about the high levels of nuclear IL-33 in baseline/uninfected brains. Does the cellular source of IL-33 change following infection? The relevance of this study is emphasized with data from human tissue demonstrating similar IL-33 nuclear localization. A minor point: the tissue is described as ‘healthy human’ – a rarity – more specifically is the human Toxoplasma seropositive or negative?

To corroborate our findings that astrocytes express IL-33 in infected brain tissue, we included discussion in **lines 127-129 on page 6** of literature which has detected IL-33 in astrocytes on the RNA and protein level, including Zhang et al., Vainchtein et al., and Gadani et al. in normal mouse brain tissue^1-3^. Based on the work of Vainchtein et al., astrocytes are the predominant source for IL-33 during development, before myelinating oligodendrocytes develop and become major producers of IL-33. Furthermore, Gadani et al. has done a thorough characterization of IL-33 expression in adult brain tissue at baseline using astrocyte reporter mice. Gadani et al. showed similar frequencies of IL-33+ astrocytes and oligodendrocytes, to our data in the infected brain (Figure 1A and Figure 1B), indicating that the sources of IL-33 do not change with infection. We included discussion of these points **in lines 131-136 on page 6**. We hypothesize that the slight increase in IL-33 expression with infection (Figure S1A) is likely due to either an increase in IL-33 expression per cell, or due to turnover of IL-33-expressing cells in *T.gondii*-infected brain tissue.

Last, we agree with the reviewer that “healthy” human tissue can be a vague term. Because a large portion of the human population is seropositive for *T. gondii* and is never diagnosed^4-6^, we cannot be certain that the “healthy” human brain samples used in our study were obtained from patients seronegative for *T.gondii*. However, we do know that these were not patients whose definitive cause of death was Toxoplasmic encephalitis. This is now clarified in the **Figure S1C and D figure legend, in lines 1014-1015 on page 41**.

3. Acute versus chronic: In Figures 2 and 3 flow cytometry data demonstrate defects in T cell and monocyte recruitment. Such defects could be due to a failure to recruit activated and expanded peripheral cells or it could be a failure of activation and expansion in the periphery. Although this is acknowledged there could be additional data to underline the importance of CNS IL-33 in this peripheral immune cell recruitment. There are defects in T cell numbers in the blood and increases in T cells in the peritoneum at day 10 possibly suggesting a delay in recruitment/clearance. Perhaps serum IL-12/IFN-g could establish a normal kinetics? Or are T cells equivalently activated and expanded during acute infection? As this study is filling in gaps about initial recognition of infection in the brain (and in the absence of a recrudescent model) some data generated earlier during infection may be useful and could identify those initial sensing moments occurring in the CNS perhaps at the vasculature.

We thank the reviewer for their insight, since impacts of IL-33 signaling during acute infection can influence brain immunity to *T. gondii* and are important to consider. To address this question, we assessed serum IFN-γ levels at two acute timepoints, day 7, and day 12, in infected wildtype and ST2-deficient animals (**Figure S4 G and H**). We observed that while day 7 levels of plasma IFN-γ were unaffected by a lack of IL-33 signaling, IFN-γ was significantly reduced in day 12 serum in the absence of IL-33 signaling **(Figure S4 G and H)**. As suggested, we also assessed IL-12 levels at day 7, since IL-12 is one component of inducing early IFN-γ production, and found that IL-12 levels were not impacted by IL-33 signaling **(Figure S4I)**. Therefore, IL-33 appears to impact IFN-γ levels during the late acute/early chronic stage of infection (day 12), whereas IL-12 and IFN-γ levels are equivalent at day 7. These results are now discussed **in lines 212-214 on Page 9.**

Although IL-33 is important for IFN-γ production early in infection, there appears to be enough IFN-γ produced early during infection to control infection in the brain, since parasite burden is unaffected at day 12 in the brain **(Figure S3A)**, and IFN-γ + T cell numbers are intact in the brain at day 12 **(Figure S6C)**. This may be because early IFN-γ levels at day 7 post-infection are intact, at levels approximately 20-fold higher than at day 12, when IFN-γ is affected by IL-33 signaling **(Figure S4G and S4H)**. There is also evidence that IL-33 supports T cells specifically within brain tissue as infection progresses, and that defects during chronic infection are not solely a result of defective activation during acute infection. Numbers of IFN-γ producing T cells in the brain progressively rely on IL-33 signaling with time, decreasing compared with WT controls from day 12 post-infection, when T cells can first be detected in the brain **(Figure S6C)**, to day 21 **(Figure S6H)**, and continue to show increased dependence on IL-33 signaling by day 28 (Figure 2F). Additionally, chronic T cell activation is at least partially dependent on astrocyte expression of the IL-33 receptor (Figure 6D), further demonstrating that IL-33 signaling supports T cell responses in the brain specifically. Discussion of these results can be found in **lines 213-220 on pages 9 and 10**.

*Note: ST2^-/-^ has been changed to its gene name, *il1rl1*^-/-^, throughout the manuscript per reviewer suggestion. ST2^-/-^ is still used in this document for continuity of addressing reviews.*

Reviewer #2:

1. Proof that astrocytes are the key cell type that expresses ST2 is lacking. The only analysis of cell-type specificity of ST2 is buried in supplementary fig 6, which is a cell-sorting experiment that demonstrates that gene expression is higher in CD11b+ cells in naïve mice and very low in astrocytes. Then post-infection, expression levels are reversed. There is a nice discussion of why this might be in the paper’s discussion. But I didn’t see any data on other cell types that might express ST2, or in situ or immunostaining to look at its cell-type specific expression during chronic Toxoplasma infection. This is also critical because Gfap-cre lines frequently have some or a lot of spill-over of expression into neurons and other cell types in the brain. Thus, some validation of the Gfap-cre, floxed ST2 mice to demonstrate the degree of knockout of the gene as well as the cell-specificity of ST2 expression during Toxoplasma infection would greatly strengthen the paper.

We thank the reviewer for bringing up this important point. ST2 expression has been a major focus of ours and the single-largest challenge throughout this project, due to the large number of cells that could express ST2, and technical challenges in detecting ST2. Based on existing IL-33 literature, immune cells such as ILC2s, regulatory T cells, mast cells, and others, express the highest levels of ST2 by flow cytometry and are critical for initial responses to infection, asthma, and allergy^7-10^. To do a thorough characterization of which immune cells express ST2 during brain *T. gondii* infection, we performed flow cytometry on naïve and infected mice, finding little to no ST2 expression on effector T cells, or monocyte-derived macrophages, populations that are highly represented in the *T. gondii*-infected brain **(S9 Fig, Figs 2 and 3**). We did note ST2 expression on ILC2s in naïve brain meninges tissue, although the percentage of ILC2s present upon infection in the brain decreased significantly **(S9 Fig)**. We also found subsets of ST2+ mast cells and ST2+ regulatory T cells **(S9 Fig)**. However, our chimera data **(Fig 5)** suggests that ST2 on immune cells is not necessary to maintain critical immune cell populations or control parasite burden in the brain.

In terms of brain resident cell types, the two most documented cell types reported to express ST2 are microglia at baseline, and astrocytes during disease^1-3,11-13^. Oligodendrocytes, neurons, and endothelial cells have not been consistently reported to express ST2. We cannot rule out a potential contribution from these cell types, however, since a fraction of neurons specifically within the hippocampus were shown very recently to express ST2 on the transcript level^12^, and endothelial cells in other tissues and in culture have been shown to express ST2^14,15^. Unfortunately, despite thorough testing, we have not found an antibody for either flow cytometry or immunohistochemistry that works well to detect ST2 expression on brain resident cells (using microglia as a control) to either confirm or deny ST2 expression on endothelial cells, neurons, or oligodendrocytes, at the protein level during our infection. Furthermore, these cell types are not easily dissociated and sorted from the adult mouse brain during infection in appreciable numbers for flow cytometry or qPCR analysis. The majority of published reports have relied on PCR from isolated cells, RNA sequencing, or *in situ* hybridization to detect ST2 in the brain, the majority of which find ST2 expression almost exclusively on microglia in the healthy brain^2,3,12,13,16^. While we cannot completely rule out a contribution of other ST2+ brain resident cell types during our infection, we did attempt to avoid off-target effects of GFAPcre mice on other neural progenitor cell types by using the GFAPcre 77.6 mice (Jackson ID Stock No. 024098), which are reported to be much improved over GFAPcre 73.12 mice (Jackson Stock No. 12886) in impacting other neural progenitor cells and should reduce off target effects. We have included statements acknowledging the possibility of contribution of ST2 on other brain-resident cell types **in lines 362-368 on page 15, and lines 464-467 on pages 19 and 20.**

To address knockdown of ST2 in astrocytes, we magnetically enriched brain samples for astrocytes and performed quantitative PCR, finding an approximate 70% reduction in ST2 mRNA in GFAPcre+ animals, now included as **Figure S10B**. We hypothesize that a partial knockdown could be due to incomplete GFAP positivity in the astrocyte population upon infection.

*Note: ST2^-/-^ has been changed to its gene name, *il1rl1*^-/-^, throughout the manuscript per reviewer suggestion. ST2^-/-^ is still used in this document for continuity of addressing reviews.*

2. Is there any change in chemokine expression in the ST2 knockout mice prior to infection? These controls would help to determine if this is solely a defect in response to infection.

To address this question, we purified RNA from uninfected WT and ST2-deficient mouse brains and tested them for differences in *ccl2* at baseline by quantitative PCR, finding that *ccl2* was unchanged between groups (**Figure S7A**). Because *ccl2* is not expressed highly in the uninfected brain, and is upregulated approximately 150-fold upon infection, we expect that the dependence of *ccl2* expression on IL-33 signaling occurs solely during infection. This result is included in **lines 303-304 on page 13**.

*Note: ST2^-/-^ has been changed to its gene name, *il1rl1*^-/-^, throughout the manuscript per reviewer suggestion. ST2^-/-^ is still used in this document for continuity of addressing reviews.*

3. The data on the global knockouts of IL33 and ST2 demonstrate quite clearly that there is less inflammation and more parasites. However, there is no functional readout to say whether this is disadvantageous to the mice or not. One could imagine that actually less of an immune response is advantageous in a situation where the parasites do not grow further. Ideally, there would be some data on brain function and/or longitudinal effects on infectious burden, however if this is not possible due to lab shutdown, then the concept that either inflammation or parasite burden could be harmful should be discussed.

As the reviewer astutely points out, there are situations during *Toxoplasma gondii* infection, in the acute and chronic stages, where the immune response can be harmful if unchecked, causing immunopathology - such as IL-10 depletion^17-19^. To the reviewer’s point, we do think reduced inflammation in the absence of IL-33 signaling could be beneficial to host tissue, since ST2-deficient mice are protected during acute infection from weight loss, early mortality, and intestinal pathology (Ryffel et al.)^20^. It is possible that ST2^-/-^ mice are also protected from immunopathology in the brain during chronic infection, but with the *T. gondii* strain and infectious dose used in our studies, wildtype mice survive without widespread necrosis in the brain. This is now discussed in **lines 221-227 on Page 10.**

It is also possible, as the reviewer posits, that parasite load may not exponentially increase with time and may not be detrimental to brain tissue in ST2^-/-^ mice. This is reflected in the fact that ST2^-/-^ mice do not succumb to infection **(lines 183-184 on page 8)**. We anticipate that while IL-33 is important for controlling parasite burden, it is one component of several innate immune mechanisms utilized in the brain for sensing parasite and initiating immunity. For example, our lab has observed a similar phenotype in IL-1R^-/-^ mice^21^ to ST2^-/-^ mice – thus, we anticipate that knocking out multiple alarmin pathways at the same time would be required to cause significant destruction to brain tissue.

*Note: ST2^-/-^ has been changed to its gene name, *il1rl1*^-/-^, throughout the manuscript per reviewer suggestion. ST2^-/-^ is still used in this document for continuity of addressing reviews.*

Reviewer #3: Major deficiencies:
1. The reliance on a single data point during T. gondii infection precludes deciphering the key cellular events regulated by IL-33. More specifically, it was not clear whether the deficiency in T cells resulted in reduced recruitment and activation of monocytes (measured by iNOS). Alternatively, impaired recruitment of CCR2+ monocytes may be responsible for the incomplete activation of T cells. This part of the manuscript was not fully developed and detailed kinetics would allow the authors to establish clearer associations between IL-33, T cells, and monocytes. This reviewer does not expect that the authors will deplete the cells in question as this would fatally compromise host defense against the parasite; instead, a detailed kinetic analysis of monocyte and T cell recruitment mediated by IL-33 would fully address this question.

We thank the reviewer for bringing up this point since we are also very interested in understanding the kinetics of how IL-33R deficiency impacts immunity to *T. gondii* in the brain. To address this question, we assessed numbers and function of immune cells in the brain at day 12 post-infection, when immune cells first reach the brain in appreciable numbers, and at day 21 post-infection, during early chronic infection. We observed that at day 12 post-infection, the immune response to *T. gondii* was largely intact, with only mild deficiencies in MHCII MFI of microglia/mature macrophages, and in the frequency of T-cell IFN-γ (new **Figure S6A and B**). These results suggest that T cell activation is one of the first parameters to be affected by a lack of IL-33 signaling. At day 21, T-cell derived IFN-γ frequency and number are further decreased from wildtype mice **(Figure S6G and and S6H)**, and numbers of infiltrating myeloid cells begin to decrease, which may be a result of reduced IFN-γ-induced chemokine and adhesion factor expression, or may be uncoupled, since IL-33 can also signal on brain resident cells directly to impact immune cell recruitment (Figures 5 and 6). Interestingly, the frequency of iNOS expression in infiltrating myeloid cells was not affected at either timepoint (Figure S6E and J), indicating that iNOS deficiencies may be the phenotype to appear at the latest timepoints in ST2-deficient mice, since it is significantly reduced by day 28 post-infection (Figure 3F-1). These results are now discussed in the manuscript **in lines 263-274 on pages 11 and 12.**

*Note: ST2^-/-^ has been changed to its gene name, *il1rl1*^-/-^, throughout the manuscript per reviewer suggestion. ST2^-/-^ is still used in this document for continuity of addressing reviews.*

2. The data suggest that astrocytes are the producers of both IL-33 and CCL2 as the later mediator was triggered in an ST2-dependent manner, a signaling event seen in astrocytes; however, further examining whether astrocytes are indeed co-producers of these factors would significantly bolster the author’s findings. This reviewer feels the authors have already developed the essential experimental tools to determine if astrocytes are indeed co-producers of these soluble mediators.

To address this point, we imaged the brains of infected *ccl2*-RFP reporter mice and co-stained for IL-33 and GFAP to mark astrocytes. We found clear examples of astrocytes co-expressing nuclear IL-33 and *ccl2* (**Figure S7B**), although only approximately 25% of *ccl2+* astrocytes also expressed IL-33 (**Figure S7C**). Thus, although astrocytes can be co-producers of IL-33 and *ccl2*, IL-33 expression is not required for *ccl2* expression within the same cell. Thus, we hypothesize that *ccl2* expression occurs downstream of extracellular IL-33 signaling, for which there is precedent in the literature^7-9^. This result is mentioned **in lines 304-306 on page 13.**

**Part III – Minor Issues: Editorial and Data Presentation Modifications**
Please use this section for editorial suggestions as well as relatively minor modifications of existing data that would enhance clarity.

Reviewer #1: the data are well presented

Reviewer #2: 1. Cell-specificity of IL33. The data shows that oligodendrocytes have at least as much IL33 expression as astrocytes, and likely more in the white matter. This should be acknowledged, ie that they both make it but are signaling through astrocytes alone. The role of oligodendrocytes is presented in the paper but is not reflected in the title and other places in the text.

We have changed the text within the results and discussion to reflect that IL-33 can be expressed by both oligodendrocytes and astrocytes, but then specifically signals on astrocytes once released, **in lines 322-324 on page 14, lines 364-368 on page 15, and lines 422-423 on page 18.**

2. Similarly, the last line of the abstract implies that without astrocytic ST2 there is no adaptive immune response. This seems like an overstatement, the data appear to show that there is a reduced, inadequate response.

This language has been edited in **lines 46-47 on Page 2.**

3. In lines 309-310, the authors claim IL-33-ST2 signaling on astrocytes affects infiltrating myeloid cell numbers but Fig 6D doesn’t show that. This should be edited, and also highlighted in the discussion.

We edited the text in **line 378 on page 16 and line 463 on page 19** to state that myeloid cells are not significantly impacted by ST2-deficiency in astrocytes, and we discuss possible explanations for this finding in **lines 463-470 discussion on** **Pages 19 and 20.**

4. In the abstract, the authors state that “IL-33 signaling promotes chemokine expression within brain tissue and is required for the recruitment of peripheral anti parasitic immune cells, including IFN-g-expressing T cells and iNOS-expressing monocytes.” However they also show deficits in T cell proliferation, so it is not clear whether the decreased number of T cells is due to decreased recruitment, proliferation, or both. This sentence should be rephrased, and it would be appropriate to mention that it might be either mechanism in the discussion.

This language has been changed in **line 42** within the abstract. We further discussed this point in **lines 197-199 on page 9**, also including the possibility that IL-33 could be stimulating survival of immune cells.

5. Fig 5 is a little confusing. The diagram at the top could be utilized to make the labels in the graphs below more clear. Specifically it’s hard to figure out which is the BM and which is the host. Also the methods make it clear that SJL mice were not used but rather a congenic B6 strain carrying the Ptprc allele of SJL. SJL mice, and actually even sometimes the congenics, can have different immune responses.

We apologize for the lack of clarity and edited the schematic in Figure 5 to have the notation BM 🡪 recipient, with the four chimeric groups labeled in the same notation. We thank the reviewer for their edit on the wildtype mouse strain used, we have now edited the labels as “WT CD45.1” and “ST2^-/-^ CD45.2.”

6. Please list the antibodies used for immunohistochemistry, their sources, and their dilutions. Also, please clarify - was the IL33 actually detected by in situ or was it detected by immunohistochemistry in Figs 1 and S1? It looks so nuclear and the figure legends refer to “IL33 expression” but I didn’t see this in the methods.

We apologize for this oversight. We made a resources table with all catalog numbers, and forgot to include it in the original submission. All catalog numbers, dilutions, and staining methodology have been added to the methods and figure legend of Figs 1 and S1.

7. For Fig. 1B the authors should state their Ns for quantification in the figure legend.

We have edited figure 1B to show data points from individual mice, with an n of 5 animals.

8. Line 156 needs the reference “Figure 1E.”

We have now included the reference in **line 171 on Page 8.**

9. Given that there is no iNOS expression in the periphery in both WT and ST2-/- infected mice, is there any expression in naïve mice? Or if iNOS expression has been observed in the periphery in other infection models, it could be worth mentioning to draw distinctions from Toxoplasma gondii’s mechanism of infection.

iNOS expression is a mechanism used to control a range of intracellular pathogens^22,23^, and is not highly expressed in naïve tissue. Its expression has been observed at many of sites of infection, such as the lung, intestine, spleen, and elsewhere in bacterial, and other parasitic infections^22,23^. We have observed iNOS to be expressed in the peritoneum during acute infection, but at much lower frequencies than in the brain during chronic infection (5-10% versus 30-40%, Figure S5, Figure 3). In addition to increased iNOS expression in the brain compared with other tissues, iNOS is important for survival of chronic infection, since global iNOS knockout mice do not succumb to infection until 24DPI, when the parasite has established a chronic infection in the brain^24^. Why iNOS is indispensable during acute *T. gondii* infection, while in other infections it is not, is not well understood. We hypothesize that other mechanisms of host resistance are employed during acute infection that can compensate for or outweigh iNOS expression, which do not occur during chronic infection. A discussion of iNOS expression is included **in lines 254-258 on page 11.**

10. Fig 5E-G might be more clear if only the significant relationships are annotated in the bar graphs, and if there were more space over the graph to separate the multiple comparisons.

We have made these suggested changes to Figure 5E-G.

11. In Fig 6B and 6E, it’s interesting that microglia and astrocytes have reverse trends for CD4+ cell numbers. In the Cx3cr1-Cre constitutive KO, this is the only phenotype that is affected, whereas in the Gfap-Cre constitutive KO, it is one of the only phenotypes that isn’t affected. This makes sense since there is a connection between CD4+ T cells and MHC II expressing microglia, so perhaps it could be worth commenting on.

We are also very interested in antigen presentation as a possible mechanism brain resident cells use to support immunity downstream of IL-33 signaling. We added discussion of this concept in **lines 380-382 of page 16.**

12. Line 356 missing citation for in vitro experiment.

These citations have been added, now in **line 429 on page 18.**

13. Line 368 missing citation for microglia.

We added a citation showing downregulation of microglia inflammatory genes during *T. gondii* infection, **in lines 441-442 on Page 19.**

Reviewer #3: Minor comments:

1. ST2 KO mice should be abbreviated as Il1rl1 KO

We have changed ST2^-/-^ to *il1rl1*^-/-^ throughout the manuscript.

2. The significance of the manuscript will be improved if the authors can establish a mechanism of IL-33 release in response to T. gondii infection. It was not clear whether the infected cells or neighboring cells release this alarmin during the parasitic infection.

We are also very interested in whether or not IL-33 is released upon direct infection of a cell, or from bystander inflammation. Unfortunately, we do not currently have the fluorescent astrocyte or oligodendrocyte reporter mice, or reporter *T. gondii* strain needed to visualize parasite infection, or lack of infection– to watch cell death in real time and determine whether or not the dying cell is infected. We can, however, hypothesize that functional IL-33 is released upon necrotic cell death, since caspases involved in apoptotic and pyroptotic cell death inactivate IL-33^7,25,26^.

1	Vainchtein, I. D. *et al.* Astrocyte-derived interleukin-33 promotes microglial synapse engulfment and neural circuit development. *Science* **359**, 1269-1273, doi:10.1126/science.aal3589 (2018).

2	Zhang, Y. *et al.* An RNA-sequencing transcriptome and splicing database of glia, neurons, and vascular cells of the cerebral cortex. *J Neurosci* **34**, 11929-11947, doi:10.1523/JNEUROSCI.1860-14.2014 (2014).

3	Gadani, S. P., Walsh, J. T., Smirnov, I., Zheng, J. & Kipnis, J. The glia-derived alarmin IL-33 orchestrates the immune response and promotes recovery following CNS injury. *Neuron* **85**, 703-709, doi:10.1016/j.neuron.2015.01.013 (2015).

4	Pappas, G., Roussos, N. & Falagas, M. E. Toxoplasmosis snapshots: global status of Toxoplasma gondii seroprevalence and implications for pregnancy and congenital toxoplasmosis. *Int J Parasitol* **39**, 1385-1394, doi:10.1016/j.ijpara.2009.04.003 (2009).

5	Hunter, C. A. & Sibley, L. D. Modulation of innate immunity by Toxoplasma gondii virulence effectors. *Nat Rev Microbiol* **10**, 766-778, doi:10.1038/nrmicro2858 (2012).

6	Hill, D. & Dubey, J. P. Toxoplasma gondii: transmission, diagnosis and prevention. *Clin Microbiol Infect* **8**, 634-640, doi:10.1046/j.1469-0691.2002.00485.x (2002).

7	Liew, F. Y., Girard, J. P. & Turnquist, H. R. Interleukin-33 in health and disease. *Nat Rev Immunol* **16**, 676-689, doi:10.1038/nri.2016.95 (2016).

8	Liew, F. Y., Pitman, N. I. & McInnes, I. B. Disease-associated functions of IL-33: the new kid in the IL-1 family. *Nat Rev Immunol* **10**, 103-110, doi:10.1038/nri2692 (2010).

9	Martin, N. T. & Martin, M. U. Interleukin 33 is a guardian of barriers and a local alarmin. *Nat Immunol* **17**, 122-131, doi:10.1038/ni.3370 (2016).

10	Molofsky, A. B., Savage, A. K. & Locksley, R. M. Interleukin-33 in Tissue Homeostasis, Injury, and Inflammation. *Immunity* **42**, 1005-1019, doi:10.1016/j.immuni.2015.06.006 (2015).

11	Yang, Y. *et al.* ST2/IL-33-Dependent Microglial Response Limits Acute Ischemic Brain Injury. *J Neurosci* **37**, 4692-4704, doi:10.1523/JNEUROSCI.3233-16.2017 (2017).

12	Nguyen, P. T. *et al.* Microglial Remodeling of the Extracellular Matrix Promotes Synapse Plasticity. *Cell* **182**, 388-403 e315, doi:10.1016/j.cell.2020.05.050 (2020).

13	Yasuoka, S. *et al.* Production and functions of IL-33 in the central nervous system. *Brain Res* **1385**, 8-17, doi:10.1016/j.brainres.2011.02.045 (2011).

14	Cao, K. *et al.* IL-33/ST2 plays a critical role in endothelial cell activation and microglia-mediated neuroinflammation modulation. *J Neuroinflammation* **15**, 136, doi:10.1186/s12974-018-1169-6 (2018).

15	Gautier, V. *et al.* Extracellular IL-33 cytokine, but not endogenous nuclear IL-33, regulates protein expression in endothelial cells. *Sci Rep* **6**, 34255, doi:10.1038/srep34255 (2016).

16	Fairlie-Clarke, K. *et al.* Expression and Function of IL-33/ST2 Axis in the Central Nervous System Under Normal and Diseased Conditions. *Front Immunol* **9**, 2596, doi:10.3389/fimmu.2018.02596 (2018).

17	Wilson, E. H., Wille-Reece, U., Dzierszinski, F. & Hunter, C. A. A critical role for IL-10 in limiting inflammation during toxoplasmic encephalitis. *J Neuroimmunol* **165**, 63-74, doi:10.1016/j.jneuroim.2005.04.018 (2005).

18	Gazzinelli, R. T. *et al.* In the absence of endogenous IL-10, mice acutely infected with Toxoplasma gondii succumb to a lethal immune response dependent on CD4+ T cells and accompanied by overproduction of IL-12, IFN-gamma and TNF-alpha. *J Immunol* **157**, 798-805 (1996).

19	Neyer, L. E. *et al.* Role of interleukin-10 in regulation of T-cell-dependent and T-cell-independent mechanisms of resistance to Toxoplasma gondii. *Infect Immun* **65**, 1675-1682, doi:10.1128/IAI.65.5.1675-1682.1997 (1997).

20	Ryffel, B. *et al.* Blockade of IL-33R/ST2 Signaling Attenuates Toxoplasma gondii Ileitis Depending on IL-22 Expression. *Front Immunol* **10**, 702, doi:10.3389/fimmu.2019.00702 (2019).

21	Batista, S. J. *et al.* Gasdermin-D-dependent IL-1alpha release from microglia promotes protective immunity during chronic Toxoplasma gondii infection. *Nat Commun* **11**, 3687, doi:10.1038/s41467-020-17491-z (2020).

22	Chakravortty, D. & Hensel, M. Inducible nitric oxide synthase and control of intracellular bacterial pathogens. *Microbes Infect* **5**, 621-627, doi:10.1016/s1286-4579(03)00096-0 (2003).

23	James, S. L. Role of nitric oxide in parasitic infections. *Microbiol Rev* **59**, 533-547 (1995).

24	Scharton-Kersten, T. M., Yap, G., Magram, J. & Sher, A. Inducible nitric oxide is essential for host control of persistent but not acute infection with the intracellular pathogen Toxoplasma gondii. *J Exp Med* **185**, 1261-1273 (1997).

25	Cayrol, C. & Girard, J. P. The IL-1-like cytokine IL-33 is inactivated after maturation by caspase-1. *Proc Natl Acad Sci U S A* **106**, 9021-9026, doi:10.1073/pnas.0812690106 (2009).

26	Luthi, A. U. *et al.* Suppression of interleukin-33 bioactivity through proteolysis by apoptotic caspases. *Immunity* **31**, 84-98, doi:10.1016/j.immuni.2009.05.007 (2009).
